# Triangle Tilt Surgery in an Older Pediatric Patient With Obstetric Brachial Plexus Injury

**Published:** 2009-06-30

**Authors:** Rahul K. Nath, Abdelouahed Amrani, Sonya E. Melcher, Mitchell G. Eichhorn

**Affiliations:** ^a^ Texas Nerve and Paralysis Institute, Houston, TX 77030; ^b^ Children's Hospital of Rabat, Rabat, Morocco; ^c^ Research Division, Texas Nerve and Paralysis Institute, Houston, TX 77030

## Abstract

Children with an obstetric brachial plexus injury have an elevated risk of long-term impairment if they do not fully recover by the age of 3 months. Persistent nerve damage leads to muscle abnormalities and progressive muscle and bone deformities. Several procedures have been described to treat this severe deformity. We have demonstrated the benefits of the triangle tilt procedure in young children with a mean age of 6.4 years (2.2 to 10.3), yet the treatment of humeral head subluxation secondary to obstetric brachial plexus injury represents a challenge in older pediatric patients. This case report demonstrates the effectiveness of triangle tilt surgery for the treatment of glenohumeral joint deformity in a 12 year old pediatric patient with left sided residual brachial plexus injury. The patient in this study showed noticeable clinical improvements, an improvement in glenohumeral joint dysplasia, and a reduction in humeral head subluxation 2 years after triangle tilt surgery. There was functional improvement 25 months after surgery. The patient's total Mallet score for shoulder function improved from 14 to 20 (of 25). In this case report, we demonstrate that the triangle tilt procedure can be used for older pediatric patients without modification. This observation has provided valuable information and is, to our knowledge, the first documented improvement of a glenohumeral joint deformity in an older pediatric patient. Future studies will be needed to determine the long-term success of triangle tilt surgery in this age group.

Children with an obstetric brachial plexus injury have an elevated risk of long-term impairment if they do not fully recover by the age of 3 months.^1^ Persistent nerve damage leads to muscle abnormalities and progressive muscle and bone deformities.^2^,^3^ The most common deformities develop after damage to the upper trunk (C5–C6) of the brachial plexus, which innervates the shoulder girdle musculature.^4^ The adductors and internal rotators of the shoulder develop contractures and the abductors and external rotators are weakened. The resulting muscular imbalances cause severe bony deformities and glenohumeral joint subluxation because the growing bones and joints of an infant require the correct muscular forces for normal development.^5^ Bony deformities are most prevalent in the scapula, whose development is altered by abnormal shoulder girdle musculature. The scapula becomes elevated, protracted, and deformed in a manner that has been previously described as the SHEAR (scapular hypoplasia, elevation, and rotation) deformity.^5^ The glenoid fossa flattens and the acromion process “hooks,” causing impingement on the humeral head.^4^

The SHEAR deformity is associated with glenohumeral deformity, which has been previously described as a combination of glenoid retroversion, joint incongruity, and posterior humeral head dislocation or subluxation.^2^,^6^ The origin of humeral head subluxation is similar to the cause of the SHEAR deformity and can be traced to muscle imbalances and the inherent plasticity of the infant glenohumeral joint during development.^3^

Several procedures have been described to treat this severe deformity, including open reduction with anterior release,^7^ anterior and posterior approach,^3^ reduction combined with tendon transfers to rebalance the glenohumeral joint,^8^ arthroscopic release,^9^,^10^ humeral osteotomy,^11^ and triangle tilt surgery.^4^ Humeral osteotomy is recommended for the treatment of glenohumeral deformity in older patients^12^; however, this procedure is performed primarily to salvage function and does not improve the glenohumeral deformity or stimulate joint remodeling. There is a high incidence of recurrence following humeral osteotomy as well. Anterior capsule release improves glenohumeral alignment, but it is associated with external rotation deformities.^12^ The triangle tilt procedure was designed to improve the position of the humeral head in the glenoid, correct the impingement that occurs in the SHEAR deformity, and stimulate glenohumeral joint remodeling.^4^ The procedure consists of osteotomies of the distal clavicle and acromion, which allows the acromioclavicular triangle to “tilt” back to a more normal position.

Although a previous publication described the benefits of the triangle tilt procedure in young children with a mean age of 6.4 years (2.2 to 10.3),^4^ the usefulness of triangle tilt surgery in older pediatric patients has not been discussed. This case report demonstrates the effectiveness of triangle tilt surgery for the treatment of glenohumeral joint deformity in a 12 year old pediatric patient with residual brachial plexus injury.

## CASE PRESENTATION

**Figure 1:**
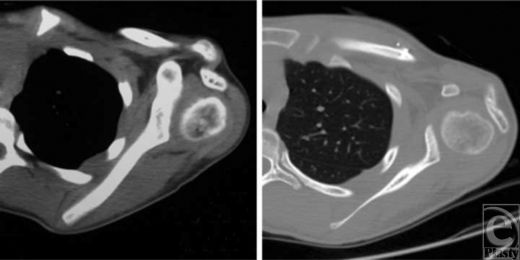
Axial computerized tomographic scans of the affected shoulder: 5.5 months before surgery (left) and 25 months after surgery (right) showing improved posterior humeral head subluxation (‐11% to 34%) and glenoid retroversion (‐53° to ‐22°). Both scans are at the level of the humeral head and have been chosen to best represent the relationship between the humeral head and glenoid.

The patient was a 12-year-old girl with left-sided obstetrical brachial plexus injury. Her injury was surgically treated at a different institution 5 years before triangle tilt surgery. The previous surgery included the release of subscapularis muscle, pectoralis major fractional lengthening, latissimus to infraspinatus tendon transfer, teres major to supraspinatus tendon transfer, and coracoacromial ligament release; there was only marginal improvement in function and no improvement in deformity following these procedures. Physical examination revealed medial rotation contracture of the shoulder with apparently unrecognized SHEAR deformity (grade 4). She could not supinate and had a flaring trumpet sign during hand-to-mouth movements. Computerized tomographic scanning revealed the extent of posterior humeral head subluxation (‐11% humeral head anterior to the scapular line, and a negative value indicates dislocation), glenoid retroversion (‐53° from perpendicular to the scapular line), and glenohumeral joint dysplasia (Fig 1).

Triangle tilt surgery was performed on the patient. The procedure began with an incision along the medial edge of the superomedial border of the scapula. Soft tissue was dissected from the scapula and the prominent superomedial angle of the scapula was excised with a bone cutter to create a rounded surface. An incision was made over the spine of the scapula and soft tissue dissection exposed the scapular spine, which was cut with an electric saw. The scapula and the acromion process derotated after osteotomy, indicating preoperative twisting and internal rotation caused by the elevated scapula. The bone removed during osteotomy was morselized and placed in the bone defect between the scapular spine and the acromion process. An incision was made over the distal third of the clavicle and the soft tissue was dissected to expose the clavicle. Osteotomy of the clavicle was performed with an electric saw and the distal and proximal clavicle segments were fastened together in semirigid fixation with titanium screws and absorbable suture. The clavicle segments were noted to “twist” relative to each other after the osteotomy, indicating torsion in the clavicle due to scapular elevation. An incision was then made above the posterior glenohumeral joint and careful dissection through the deltoid muscle exposed the posterior glenohumeral capsule. Laxity in the capsule was removed with a circumferential suture in a purse-string fashion. Wounds were closed in multiple layers and the patient was splinted in adduction and supination, with the humerus externally rotated and in a neutral position. The patient was splinted for 6 weeks and then splinted only at night for additional 3 months. Physical therapy was prescribed for 6 months.

**Figure 2:**
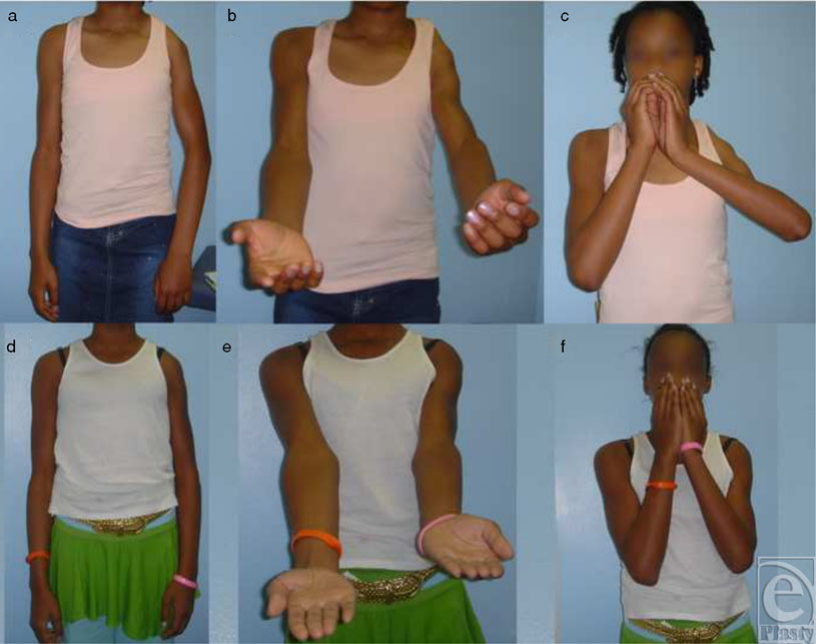
Arm appearance and shoulder function before and after triangle tilt surgery. Preoperative (a–c) images were taken the day before surgery. Postoperative (d–f) images are 5 months after surgery and show improvements that were maintained at last follow-up (25 months). Three positions are shown: resting position (a, d), supination showing improvement due to improved shoulder position (b, e), and the Mallet and hand-to-mouth movements (c, f).

There was functional improvement 25 months after surgery (Fig 2). A postoperative computerized tomographic scan taken 25 months after surgery showed an improvement in humeral head subluxation (from ‐11% to 34%), a reduction in glenoid retroversion (from ‐53° to ‐22°), and an improvement in glenohumeral joint dysplasia (Fig 1). The patient's total Mallet score for shoulder function improved from 14 to 20 (of 25); external rotation and hand-to-nose and hand-to-mouth movements each improved 2 points (of 5). Abduction was maximized by the muscle transfer surgery 5 years previously and was thus unchanged.

## DISCUSSION

A major treatment goal in children with obstetric brachial plexus injury is to obtain optimal range of shoulder motion through improved glenohumeral alignment. Shoulder deformities are very common in this patient population and these developmental abnormalities lead to poor function, pain and loss of quality of life beginning as soon as the teen years. Therefore, establishing glenohumeral congruency is a fundamental goal of most brachial plexus functional restorations. Repositioning of the glenohumeral joint to maximize alignment does not only improve function and growth, but has been shown to encourage joint remodeling.^2^,^12^ Muscle releases and tendon transfers may arrest progressive glenohumeral dysplasia and possibly influence some of the long-term effects of joint misalignment and muscle imbalance in younger patients.^2^,^11^,^12^ In older patients, these approaches remain unsatisfactory and most surgeons prefer not to treat these patients.^12^ Humeral osteotomy sometimes provides functional improvements^13^–^16^; however, it only moderately improves medial rotation contractures in mild to moderate cases and does not improve severe cases. Additionally, humeral osteotomy does not address the glenohumeral deformity, which is the major cause of morbidity in this patient population.^11^ The triangle tilt procedure provides functional improvements by directly addressing the glenohumeral deformity, and results in functional improvement as well as future remodeling of the joint.

During surgery, the distal acromioclavicular triangle returns to a more physiological position when osteotomies of the clavicle and acromion are performed, suggesting that the abnormal bony structures in the shoulder are under significant biomechanical stresses. The plane of the acromioclavicular triangle is “tilted” toward the neutral position, and the humeral head, being related to the distal structures, moves into a more normal position.^4^ Thus, glenohumeral alignment is largely restored and remodeling can begin.

The triangle tilt procedure has been previously shown as an effective treatment option for children and can be used for adolescents without modification. The procedure can theoretically be applied to patients between the ages of 8 months and 16 years. Triangle tilt is ideally suited for patients who have developed a medial rotation contracture and scapular elevation; if these abnormalities are observed, the surgery is effective in the presence of mild, moderate and severe neurological defects, including complete glenohumeral dislocations and as salvage for failed previous reconstructive surgeries. A nonunion of the clavicle could potentially occur if the clavicle segments do not heal. A complete separation of the acromion process from the clavicle is crucial for success. It is also necessary to splint the patient as described, with adduction in the scapular line, external rotation in the humerus, and supination.

## CONCLUSIONS

The treatment of humeral head subluxation secondary to obstetric brachial plexus injury represents a challenge in older pediatric patients. Triangle tilt surgery is effective in the treatment of older pediatric patients because it directly addresses the bony deformations that occur during childhood. The 12 year old patient in this study showed noticeable clinical improvements, an improvement in glenohumeral joint dysplasia, and a reduction in humeral head subluxation 2 years after triangle tilt surgery. This observation has provided valuable information and is, to our knowledge, the first documented improvement of a glenohumeral joint deformity in an older pediatric patient. Future studies will be needed to determine the long-term success of triangle tilt surgery in this age group.
